# Autosomal dominant mitochondrial membrane protein‐associated neurodegeneration (MPAN)

**DOI:** 10.1002/mgg3.736

**Published:** 2019-05-13

**Authors:** Allison Gregory, Mitesh Lotia, Suh Young Jeong, Rachel Fox, Dolly Zhen, Lynn Sanford, Jeff Hamada, Amir Jahic, Christian Beetz, Alison Freed, Manju A. Kurian, Thomas Cullup, Marlous C. M. van der Weijden, Vy Nguyen, Naly Setthavongsack, Daphne Garcia, Victoria Krajbich, Thao Pham, Randy Woltjer, Benjamin P. George, Kelly Q. Minks, Alexander R. Paciorkowski, Penelope Hogarth, Joseph Jankovic, Susan J. Hayflick

**Affiliations:** ^1^ Molecular & Medical Genetics, Pediatrics and Neurology Oregon Health & Science University Portland Oregon; ^2^ Parkinson's Disease Center and Movement Disorder Clinic, Department of Neurology Baylor College of Medicine Houston Texas; ^3^ Department of Clinical Chemistry Jena University Hospital Jena Germany; ^4^ Developmental Neurosciences, GOSH‐Institute of Child Health, UCL & Department of Neurology Great Ormond Street Hospital London UK; ^5^ North East Thames Regional Genetics Laboratory London UK; ^6^ Department of Neurology University Medical Center Groningen Groningen The Netherlands; ^7^ Pathology Oregon Health & Science University Portland Oregon; ^8^ Department of Neurology University of Rochester Medical Center Rochester New York; ^9^ Departments of Pediatrics, Biomedical Genetics, and Neuroscience University of Rochester Medical Center Rochester New York

**Keywords:** *C19orf12*, mitochondrial membrane protein‐associated neurodegeneration, MPAN, NBIA, neurodegeneration with brain iron accumulation

## Abstract

**Background:**

Mitochondrial membrane protein‐associated neurodegeneration (MPAN) is caused by pathogenic sequence variants in *C19orf12*. Autosomal recessive inheritance has been demonstrated. We present evidence of autosomal dominant MPAN and propose a mechanism to explain these cases.

**Methods:**

Two large families with apparently dominant MPAN were investigated; additional singleton cases of MPAN were identified. Gene sequencing and multiplex ligation‐dependent probe amplification were used to characterize the causative sequence variants in *C19orf12*. Post‐mortem brain from affected subjects was examined.

**Results:**

In two multi‐generation non‐consanguineous families, we identified different nonsense sequence variations in *C19orf12* that segregate with the MPAN phenotype. Brain pathology was similar to that of autosomal recessive MPAN. We additionally identified a preponderance of cases with single heterozygous pathogenic sequence variants, including two with de novo changes.

**Conclusions:**

We present three lines of clinical evidence to demonstrate that MPAN can manifest as a result of only one pathogenic *C19orf12* sequence variant. We propose that truncated C19orf12 proteins, resulting from nonsense variants in the final exon in our autosomal dominant cohort, impair function of the normal protein produced from the non‐mutated allele via a dominant negative mechanism and cause loss of function. These findings impact the clinical diagnostic evaluation and counseling.

## 1 INTRODUCTION

Neurodegeneration with brain iron accumulation (NBIA) comprises a group of inherited disorders that share the common feature of iron accumulation in the basal ganglia and present with a variety of neurologic and psychiatric manifestations (Gregory & Hayflick, 2013). The genetic bases of most NBIA disorders are now known (Meyer, Kurian, & Hayflick, 2015). *C19orf12* pathogenic sequence variants cause mitochondrial membrane protein‐associated neurodegeneration (MPAN [MIM: 614298]) (Hartig et al., 2011). MPAN typically presents in childhood or adolescence with progressive dystonia‐parkinsonism, optic atrophy, axonal motor neuronopathy, and iron deposition in globus pallidus (GP) and substantia nigra (SN) (Hartig et al., 2011; Hogarth et al., 2013). An autosomal recessive pattern of inheritance has been demonstrated in well‐characterized cases of MPAN (Hartig et al., 2011; Hogarth et al., 2013). A common Polish founder deletion, often seen in a homozygous state in Eastern European families, accounts for many published cases of MPAN, though numerous other deleterious mutations have now also been documented (Gagliardi et al., 2015; Hartig et al., 2011; Hartig, Prokisch, Meitinger, & Klopstock, 2013).

We have observed a large number of typical MPAN cases with heterozygous pathogenic sequence variants identified in *C19orf12* in our large International Registry of NBIA and Related Disorders. Recent ascertainment of two multi‐generation families with MPAN and a heterozygous *C19orf12* pathogenic sequence variant segregating in an autosomal dominant pattern suggests that a single mutant allele is sufficient to cause the MPAN phenotype in specific cases. In two additional families, identification of heterozygous *de novo* pathogenic sequence variants causing MPAN lends further support to this concept. *De novo* pathogenic dominant variants in *C19orf12* may account for the preponderance of heterozygous cases in our repository.

## 2 MATERIALS AND METHODS

### 2.1 Ethical compliance

All subjects were consented as part of OHSU's IRB‐approved protocol e7232 with additional work covered by protocol e144 (conforming to the US Federal Policy for the Protection of Human Subjects).

### 2.2 Subjects

Individuals presented are subjects in the OHSU International NBIA Repository, a database and sample repository of more than 800 families established 28 years ago. The proband from Family 18 was originally submitted to the repository by the Parkinson's Disease Center and Movement Disorders Clinic (PDCMDC) at Baylor College of Medicine, Houston, Texas in 1995. Family 18 has been investigated periodically since that time, and medical records and samples have been collected when possible. Subject 18**‐**307 was evaluated in the PDCMDC, and subject 18**‐**411 was evaluated in the PDCMDC and separately on a research basis by the OHSU team. Family 748 was originally ascertained through the Department of Neurology, University of Rochester Medical Center, New York. In addition, the repository was queried for all subjects with single heterozygous *C19orf12* sequence variants. For some cases, clinical molecular genetic testing results were also banked in the repository.

### 2.3 Sequencing

Genomic DNA was extracted from blood, saliva, or established cell lines, and *C19orf12* exons 1–3 (NM_001031726.3) were amplified by PCR. Products were sequenced at the Vollum DNA Sequencing Core at OHSU, and variants were identified on chromatograms. In some cases, commercial exome sequencing was performed for clinical purposes and obviated research sequencing.

### 2.4 Multiplex ligation‐dependent probe amplification

A total of five *C19orf12*‐specific probes targeting the promoter (*n* = 1), the coding exons (*n* = 2), the 3'UTR (*n* = 1), and a region approximately 1 Kb downstream of the gene (*n* = 1) were utilized for multiplex ligation‐dependent probe amplification (MLPA) analysis by the Beetz Lab (Dept of Clinical Chemistry, Jena University Hospital, Germany). Five reference probes were directed against regions on distinct other chromosomes. MLPA probe design followed the guidelines provided by MRC‐Holland (The Netherlands) at www.mlpa.com. The corresponding synthetic oligonucleotides were purchased from MWG‐Eurofins (Ebersberg, Germany). MLPA reactions utilized reagents from MRC‐Holland. Analysis of data was done as described previously (Beetz et al., 2006).

### 2.5 Analysis of postmortem brain tissue

Autopsy and neuropathologic evaluation of two subjects (18**‐**306 and 748**‐**301) were performed as previously described for a case of recessive MPAN (Hogarth et al., 2013). In brief, brain tissue was immersed in 10% neutral buffered formalin for at least 10 days, followed by dissection into sections containing the midbrain, frontal cortex, hippocampus and basal ganglia. Sections were embedded in paraffin and cut into 6‐micron sections using standard methods. Sections were stained with hematoxylin and eosin, and immunohistochemistry with antibodies to alpha‐synuclein (LB509, Thermo Scientific, Rockord, IL), tau (PHF‐1, a kind gift of Dr. Peter Davies, Albert Einstein University of Medicine, Manhasset, NY), beta‐amyloid (4G8, BioLegend, San Diego, CA), ubiquitin, and TDP‐43 (Proteintech Group, Rosemont, IL) was performed after deparaffinization and prepared for antigen retrieval by 95% formic acid for 5 min and 30 min in citrate buffer (pH 6.0 at 90°C). Tissue sections were blocked with 5% nonfat dry milk in phosphate‐buffered saline and incubated with antibodies at concentrations recommended by the suppliers followed by color development using diaminobenzidine.

## 3 RESULTS

### 3.1 Evaluation of family 18

**Figure 1:**
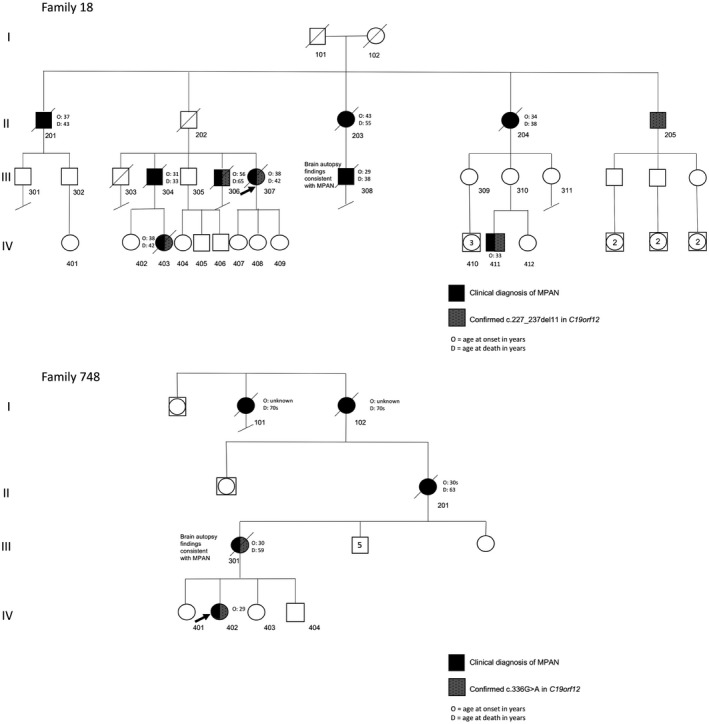
Pedigrees. Family 18: According to report by living family members, individuals 101, 102, and 202 all died in their sixties from health issues unrelated to MPAN; they were not reported to have an MPAN phenotype or signs of parkinsonism prior to their deaths. Individual 205 is 81 years and reports being in good health for his age with no signs of MPAN or parkinsonism. Family 748: Individuals 101, 102 and 201 were all reported to have had early dementia. MPAN, membrane protein‐associated neurodegeneration

The proband, a 39‐year‐old woman (18**‐**307, Figure 1) presented to the PDCMDC with an 18‐month history of neurologic symptoms. On examination, she had hypomimia, hypophonia, and left‐sided predominant parkinsonism with rigidity and bradykinesia. Magnetic resonance imaging (MRI) revealed markedly decreased signal in GP and SN bilaterally on T2‐weighted images, consistent with brain iron accumulation. The autopsy, performed at age 42 in 1998, showed rusty brown discoloration of the GP and SN. Microscopic examination revealed perivascular iron pigment deposition in the GP, gliosis, and prominent, ubiquitin‐positive axonal spheroids. The SN showed reduced number of cells, gliosis, axonal spheroids and Lewy bodies. Axonal spheroids were also present in the hippocampus and cerebellum. Her autopsy diagnosis was “Hallervorden‐Spatz disease.” The case was submitted to the OHSU repository. After initial improvement with dopaminergic medications, her parkinsonism markedly worsened over two years with development of psychosis. She died in hospice at the age of 42 years. At the time of ascertainment, she reported an extended history of similar disease in several family members suggesting of an autosomal dominant pattern.

The brother of the proband (18**‐**304, Figure 1) was published in a 1989 case report that suggested the family's disease, then called Hallervorden‐Spatz syndrome, could be the first autosomal dominant instance of the disorder (Morphy, Feldman, & Kilburn, 1989). He had progressive parkinsonism, depression, and dementia; no MRI was performed. At that time, two paternal aunts and a paternal uncle had died after similar courses, and one of the cases was confirmed by autopsy (Morphy et al., 1989). The subject died after a rapid, 8‐month decline from pulmonary edema just before his 34th birthday.

**Figure 2:**
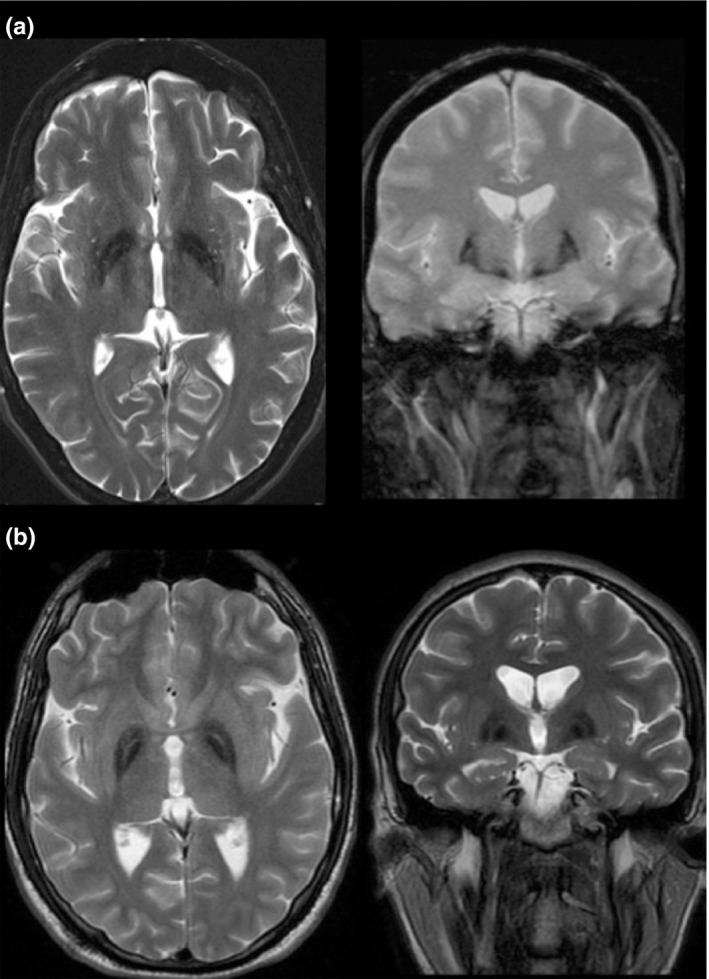
MRI findings in dominant MPAN. T2‐weighted imaging of brain at the level of the globus pallidus (axial left; coronal right) showing hypointense signal indicative of iron accumulation in subject 18‐411 (panel a, presumed dominant case) and subject 248 (panel b, recessive case). Characteristic of MPAN and seen in both cases is the preservation of isointense signal in the medial medullary lamina, which localizes between the globus pallidus interna and externa. MPAN, membrane protein‐associated neurodegeneration; MRI, Magnetic resonance imaging

Subject 18**‐**411 (Figure 1, Video S1), a 34 year‐old previously healthy employed male who was also a competitive athlete, presented in 2016 with a one‐year history of gait imbalance. His symptoms progressed rapidly to marked motor slowness and shuffling gait, tremors in both arms, and anxiety. Neurological examination showed hypomimia, hypokinetic dysarthria and hypophonia, moderate right‐sided predominant bradykinesia and rigidity, bilateral postural and kinetic hand tremor, decreased right arm swing, stooped posture and shuffling gait. MRI of his brain revealed T2 hypointense signal in the GP, with isointense signal at the medial medullary lamina (Figure 2). The DaT scan was consistent with parkinsonism with reduced dopamine transporter uptake in the striatum. His parkinsonism improved markedly after initial trial of levodopa (Video S1) but he soon developed levodopa‐related motor fluctuations and dyskinesia. At the time of presentation, the subject reported a family history that we recognized as that of Family 18 from the OHSU repository. He was subsequently identified as a first cousin once removed to 18**‐**307. Updates to the family history revealed additional affected individuals. His mother (18**‐**310) was examined and did not have any clinical features concerning for dystonia or parkinsonism.

Subject 18**‐**306, a sibling to the proband, died from complications of MPAN at 65 years of age. He had mild cognitive changes starting at 55 years that progressed to dementia with parkinsonism.

Subject 18**‐**403, the daughter of individual 18**‐**304, died at 42 years of age. She developed depression at 19 years but was otherwise stable until 38. At that time, she developed gait changes and cognitive decline, followed by urinary incontinence and inability to perform activities of daily living.

Overall, nine affected individuals from three generations have been identified in family 18. Shortly after publication of the *C19orf12* gene and associated phenotype^3^, the proband's sample was sequenced on a research basis at OHSU and a novel heterozygous 11 base‐pair deletion was detected: c.227_237del (p. Met76Thrfs*3). Although the sequence variant caused a frameshift/premature stop in the protein and was predicted to be pathogenic, MPAN had only recently been described and was reported as an autosomal recessive disorder. At that time, it was considered most likely that she was a carrier of a recessive *C19orf12* variant that could not, in isolation, explain her disease. In the three additional affected individuals from whom we were subsequently able to collect samples, the *C19orf12* sequence variant was found to segregate with disease (Figure 1). Subject 18**‐**205 (80 years of age), the only surviving and healthy sibling from the first affected generation, also harbors the sequence variant. Neither his children nor his grandchildren are reported to have any clinical signs of MPAN. Similarly, neither 18**‐**101 nor 18**‐**102 were reported to have symptoms prior to their deaths. In clinically affected individuals, onset ranges from 29 to 56 years.

### 3.2 Evaluation of family 748

The proband (748**‐**402, Figure 1) is a 32 year‐old female with parkinsonism and progressive neurocognitive decline that started in her late 20s following a first trimester miscarriage. The patient noted mood changes and fatigue and was diagnosed with depression. This was followed by worsening gait instability, slowed movements, difficulty with fine motor skills, and then debilitating pseudobulbar affect and urinary incontinence. Her exam initially showed rigidity, spasticity, bradykinesia, postural instability, marked hyperreflexia, and extensor toe sign bilaterally. She developed tremor approximately a year after her initial cognitive decline. Carbidopa/levodopa lessened her rigidity, bradykinesia, and tremor. Her brain MRI showed iron accumulation in the GP and SN. Genetic testing identified a maternally inherited heterozygous *C19orf12* pathogenic sequence variant (c.336G>A, p.W112*).

Her mother (748**‐**301, Figure 1) developed personality changes, slowed movements, and cognitive decline in her 30's, after the birth of her fourth child. At 55 years she had worsening dementia and parkinsonism. A brain MRI done several years earlier had shown basal ganglia iron accumulation. Genetic testing confirmed that she harbored the heterozygous variant found in her daughter. She died at age 59 years, and her family donated her brain for research studies. The family reported similar histories of early‐onset dementia and progressive disease in the proband's maternal grandmother (748**‐**201), great‐grandmother (748**‐**102) and sister of her great‐grandmother (748**‐**101).

### 3.3 Additional cases with heterozygous pathogenic sequence variants in *C19orf12*

Since the initial identification of *C19orf12*, the OHSU International NBIA Repository has collected cases of MPAN. In total, there are 18 heterozygous cases from 13 families (Table 1) and 22 homozygous or compound heterozygous cases from 19 families (Table 2) entered in the repository. Only *C19orf12* exons and intron borders were analyzed, therefore deep intronic pathogenic sequence variants were not expected to be detected. Additional testing for deletions and duplications by MLPA failed to identify a second mutation in any of the heterozygous families. None of the parents of heterozygous singleton cases have manifest MPAN or related features. Affected siblings from family 691 share the same copy of the nonsense variant c.238C>T. These siblings were adopted, and parental disease status is unknown.

**Table 1:** Heterozygous cases from the OHSU International NBIA Repository

Subject	Gene mutation	Protein alteration	Mutation type	Del/dup testing	Age at onset	Major features
18‐306	c.227_237del11	p.Met76Thrfs*3	Frameshift‐ premature stop	Not tested (18**‐**411 negative)	55 years	Cognitive decline, parkinsonism
18‐307	c.227_237del11	p.Met76Thrfs*3	Frameshift‐ premature stop	Not tested (18**‐**411 negative)	37 years	Neuropsychiatric changes, progressive parkinsonism
18‐403	c.227_237del11	p.Met76Thrfs*3	Frameshift‐ premature stop	Not tested (18**‐**411 negative)	19 years	Neuropsychiatric changes, gait change, cognitive decline
18‐411	c.227_237del11	p.Met76Thrfs*3	Frameshift‐ premature stop	Negative	34 years	Gait imbalance, motor slowness, tremor, anxiety, progressive parkinsonism
220	c.278delC	p.Pro93Leufs*26	Frameshift‐ premature stop	Negative	18 years	Optic atrophy, progressive parkinsonism, cognitive decline
227	c.256C>T	p.Gln86*	Nonsense	Negative	12 years	Gait changes, wheelchair at 18 years, optic atrophy, cognitive decline
392	c.278dupC	p.Pro93Profs*8	Frameshift‐ premature stop	Negative	9 years	Cognitive decline, optic atrophy, dystonia and dysarthria
437	c.357dupG	p.Ala120Glyfs*32	Frameshift‐ premature stop	Negative	29 years	Neuropsychiatric changes, parkinsonism, cognitive decline
474	c.279delT	p.Ala94Profs*25	Frameshift‐ premature stop	Negative	9 years	Falling, poor school performance, dysarthria
630	c.300delT	p.Phe100Leufs*19	Frameshift‐ premature stop	Negative	5 years	Gait changes, optic atrophy, spastic paraparesis, cognitive decline
655	c.268G>T	p.Glu90*	Nonsense	Negative	22 years	Gait changes, depression, mild dystonia and dysarthria
661	c.279_282del TGCC de novo	p.Ala94Serfs*24	Frameshift‐ premature stop	Negative	4 years	Developmental delay, spasticity, dystonia, disinhibited personality
663	c.349C>T	p.Gln117*	Nonsense	Negative	18 months	Dystonia, lower limb spasticity, sensorineural hearing loss
691‐1	c.238C>T	p.Gln80*	Nonsense	Negative	10 years	Progressive spastic tetraparesis, optic disc pallor, dysphagia
691‐2	c.238C>T	p.Gln80*	Nonsense	Negative	10 years	Progressive spastic tetraparesis, cognitive decline, optic disc pallor
698	c.238C>T de novo	p.Gln80*	Nonsense	Negative	5 years	Gait disturbance, optic atrophy, neuropsychiatric symptoms
748‐301	c.336G>A	p.Trp112*	Nonsense	Not tested (748**‐**402 negative)	30 years	Neuropsychiatric symptoms, parkinsonism, dementia
748‐402	c.336G>A	p.Trp112*	Nonsense	Negative	28 years	Cognitive decline, parkinsonism, dysarthria

Note

Del/dup testing = testing for deletions and duplications by multiplex ligation‐dependent probe amplification (MLPA).

**Table 2:** Homozygous/ compound heterozygous cases from the OHSU International NBIA Repository

Subject	Gene mutation	Protein alteration	Mutation type	Del/dup testing	Age at onset	Major features
76	c.116C>T c.205G>A	p.Ser39Phe p.Gly69Arg	Missense Missense	N/A	11 years	Gait changes, cognitive decline, dystonia
160	c.204_211del11 (homozygous)	p.Gly69Argfs*10	Frameshift‐ premature stop	Negative	10 years	Tremor, cognitive decline, spastic tetraparesis, optic atrophy
165	c.204_211del11 c.157G>A	p.Gly69Argfs*10 p.Gly53Arg	Frameshift‐ premature stop; missense	N/A	10 years	Gait change, spasticity, optic atrophy
195	c.248C>T c.400G>C	p.Pro83Leu p.Ala134Pro	Missense; missense	N/A	10 years	Optic atrophy, spasticity, parkinsonism, cognitive decline
196	c.204_211del11 c.294G>C	p.Gly69Argfs*10 p.Arg98Ser	Frameshift‐ premature stop; missense	N/A	29 years	Cognitive decline, expressive dysphasia, parkinsonism
248	c.204_211del11 c.205G>A	p.Gly69Argfs*10 p.Gly69Arg	Frameshift‐ premature stop; missense	N/A	9	Dysarthria, gait change, dystonia, parkinsonism, incontinence, cognitive decline
251‐1	c.194delG (homozygous)	p.A67Lfs*6	Nonsense	Negative	13 years	Gait changes, cognitive decline, incontinance
251‐2	c.194delG (homozygous)	p.A67Lfs*6	Nonsense	251**‐**1 negative	13 years	Gait changes, cognitive decline, dysarthria
281‐1	c.142G>C c.194−2A>G	p.Ala48Pro p.?	Missense; splice site	N/A	10 years	Progressive spasticity, parkinsonism, cognitive decline
281‐2	c.142G>C c.194−2A>>G	p.Ala48Pro p.?	Missense; splice site	N/A	10 years	Developmental delay, spasticity, gait changes
334	c.157G>A c.205G>A	p.Gly53Arg p.Gly69Arg	Missense; missense	N/A	8 years	Progressive spasticity, mild intention tremor, gait changes
341	c.194G>A (homozygous)	p.Gly65Glu	Missense	Negative	4 years	Psychosis, dystonia, tremor
348	c.194G>T c.179C>T	p.Gly65Val p.Pro60Leu	Missense; missense	N/A	4 years	Spastic paraparesis, dysarthria, developmental delay
379	c.194G>T (homozygous)	p.Gly65Val	Missense	Negative	6 years	Optic atrophy, spasticity, cognitive decline
396	c.204_211del11 (homozygous)	p.Gly69ArgfsX10	Frameshift‐ premature stop	Negative	3 years	Dystonia, neuropsychiatric changes, cognitive decline, intention tremor
433	c.194G>A c.400G>C	p.Gly65Glu p.Ala134Pro	Missense; missense	N/A	9 years	Spasticity, optic atrophy, cognitive decline
438	c.204_211del11 (homozygous)	p.Gly69Argfs*10	Frameshift‐ premature stop	Negative	6 years	Spastic paraparesis, optic atrophy, dystonia, parkinsonism, cognitive decline
440	c.171_181del11 (homozygous)	p.Gly58Argfs*10	Frameshift‐ premature stop	Negative	14 years	Dysarthria, dystonia, incontinence, neuropsychiatric changes
446‐1	c.194G>T c.204_214del11	p.Gly65Val p.Gly69Argfs*10	Missense; frameshift‐ premature stop	N/A	13 years	Progressive spasticity and motor decline, optic atrophy, significant neuropsychiatric changes, cognitive decline
446‐2	c.194G>T c.204_214del11	p.Gly65Val p.Gly69Argfs*10	Missense; frameshift‐ premature stop	N/A	11 years	Progressive lower extremity spasticity, upper extremity weakness, optic atrophy, cognitive decline
447	c.94delA c.248C>T	p.Met32fs* p.Pro83Leu	Nonsense; missense	N/A	10 years	Gait change, optic atrophy, spasticity, dystonia, cognitive decline
629	c.205G>A (homozygous)	p.Gly69Arg	Missense	Negative	7 years	Developmental delay, gait change, progressive dysarthria, dysphagia and spasticity

Note

Del/dup testing = testing for deletions and duplications by multiplex ligation‐dependent probe amplification (MLPA).

N/A = no indication to do del/dup testing because two pathogenic sequence variants were identified by other methods.

Subjects with a heterozygous variant show clinical and MRI features (Figure 2) that are consistent with MPAN, and most have a phenotype that is indistinguishable from that of cases with two variants. Of note, subjects 661 and 698 were tested clinically as trios with their parents by exome sequencing, which revealed single *de novo* pathogenic sequence variants in the proband. In most other cases from the repository, parental DNA was not available.

### 3.4 Neuropathologic findings

Histologic evaluation of brain tissue from an individual from each of the two autosomal dominant families (18**‐**306 and 748**‐**301) revealed the key features previously found in recessive MPAN, including iron accumulation in the GP with profound neuronal loss, gliosis, axonal spheroids and degenerating neurons (Hartig et al., 2011; Hogarth et al., 2013). As in recessive disease, alpha‐synuclein staining was positive throughout with midbrain, limbic, and neocortical Lewy bodies and neurites in subjects with heterozygous variants. Tauopathy was limited, with pretangles and few neurofibrillary tangles, chiefly in the CA1 sector of the hippocampus. Beta‐amyloid plaques and TDP‐43‐positive inclusions were not found. No features unique to heterozygous disease were evident based on pathology previously reported in recessive cases (Hartig et al., 2011; Hogarth et al., 2013).

## 4 DISCUSSION

### 4.1 MPAN can be dominant or recessive

To date, MPAN has been documented to follow an autosomal recessive pattern of inheritance, except in a few suspect cases (Monfrini et al., 2018). We provide three lines of evidence that suggest MPAN can also be caused by a heterozygous pathogenic sequence variant in *C19orf12* on the basis of: 1) dominant inheritance exhibited by Family 18 and Family 748; 2) the manifestation of classic MPAN in 2 cases with *de novo* single mutations; and 3) a preponderance of heterozygous cases in our cohort with frameshift/premature stop or nonsense variants all clustering in the last exon. Recessive and dominant MPAN are clinically indistinguishable, with rare exceptions.

In family 748, a single pathogenic variant segregates with disease from parent to child, and two earlier generations were well‐documented to be similarly affected. In family 18, a single *C19orf12* sequence variant segregates with disease through three generations. Incomplete penetrance and variable expressivity are common phenomena in autosomal dominant single‐gene disorders and may explain the lack of symptoms in some individuals, including 18**‐**310, a presumed obligate carrier, as well as 18**‐**205 (Figure 1). This possibility invites caution in associating genotype with phenotype in dominant MPAN and will impact clinical care. In two additional families (661, 698, Table 1), we have reported the presence of a heterozygous *de novo* pathogenic sequence variant in the probands, a common observation in dominant disorders. Though we do not currently have parental DNA from the remaining heterozygous cases, we predict that most or all mutations in the remaining singletons are in fact *de novo*.

Independent of inheritance pattern, most MPAN cases are phenotypically similar. The disease manifests chiefly with gait abnormality, dystonia, dysarthria, spasticity, optic atrophy, rapidly progressive parkinsonism, cognitive decline, and psychosis. Parkinsonism initially improves with levodopa, but levodopa‐related motor fluctuations and dyskinesias are followed by gradual loss of efficacy. Correspondingly, pathologic features that were described in recessive MPAN are recapitulated in dominant disease.

Numerous examples exist of disorders caused by pathogenic sequence variants in one gene leading to different patterns of inheritance (Ben‐Shachar et al., 2009; Koch et al., 1992; Monreal et al., 1999). However, most such cases comprise phenotypically distinct disorders that were not suspected to be allelic.

### 4.2 Genotype determines inheritance pattern

In single‐gene disorders, mutation type and location influence the mechanism of disease and thus the pattern of inheritance. Some mutations cause loss‐of‐function and others gain‐of‐function. Nonsense mutations, depending on their locations within a gene, can lead to production of stable or unstable truncated protein or no protein. Nonsense‐mediated decay (NMD) is the cellular surveillance system to detect and destroy mRNA transcripts that contain a premature stop codon. But such mutations are detected only when they are located within or upstream (5′) of the penultimate exon; those occurring in the final exon or within ~50 nucleotides of the 3′ end of the penultimate exon typically escape NMD (Lewis, Green, & Brenner, 2003). We propose a plausible hypothesis for the different patterns of inheritance in MPAN based on mutation type and location within the gene.

### 4.3 Recessive MPAN

**Figure 3:**
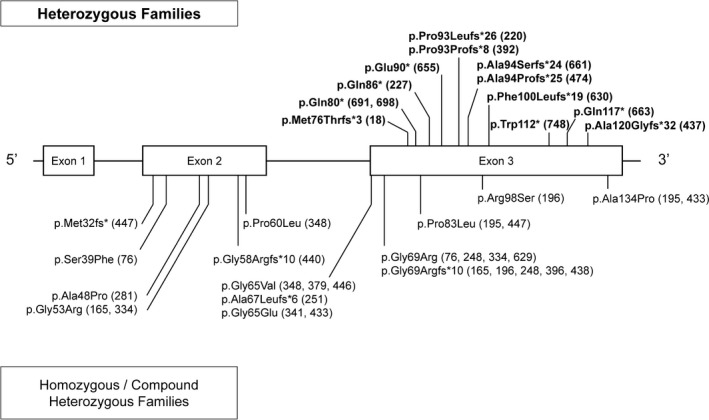
*C19orf12* mutation types. A schematic of the *C19orf12* gene with heterozygous and homozygous pathogenic sequence variants identified for all families studied. Mutant protein changes are listed first (nucleotide data are in Tables 1 and 2), followed by family numbers with each change. Single pathogenic sequence variant (dominant) cases are labeled in bold above the schematic diagram of the gene; two‐variant (recessive) cases are below

In autosomal recessive MPAN, a nonsense mutation occurring in the first or second exon would be predicted to undergo NMD, producing no protein and causing loss‐of‐function. From parental studies of recessive MPAN, we know that partial loss‐of‐function from one nonsense *C19orf12* allele causes no apparent neurologic problems in these carriers. Instead, two such mutant alleles are required to cause a complete loss‐of‐function in order to manifest disease, as we observe in our recessive cohort (Figure 3). The homozygous and compound heterozygous cases presented in Table 2 and Figure 3 comprise a mix of nonsense mutations predicted to undergo NMD, as well as missense and splice‐site mutations localized throughout the gene and predicted to cause loss‐of‐function.

### 4.4 Dominant MPAN

In contrast, 13 proposed dominant families have heterozygous nonsense variants located in the last exon (Figure 3), and all are predicted to escape NMD and produce a truncated protein. Such a mutant product could lead to disease via one of two mechanisms: the mutant protein could exhibit a toxic gain‐of‐function or it could interfere with the function of the normal protein produced from the wildtype allele, a mechanism characterized as dominant‐negative. Both of these mechanisms require only a heterozygous mutant allele to cause disease and would therefore demonstrate dominant inheritance.

### 4.5 Proposed dominant‐negative mechanism

Our clinical observations inform our hypothesis about the specific disease mechanism causing dominant MPAN. Recessive MPAN, caused by C19orf12 loss‐of‐function, is phenotypically similar to that of dominant MPAN. A toxic gain‐of‐function would be unlikely to lead to a disease phenotype that is indistinguishable from one arising from loss‐of‐function, but a dominant‐negative mechanism would ultimately cause loss‐of‐function, as in recessive disease. Based on our phenotype and genotype data, we propose a dominant‐negative mechanism in which the mutant protein interferes with the function of the normal protein encoded by the wild‐type allele, causing its loss‐of‐function.

Proteins that homo‐multimerize, such as collagen, commonly demonstrate a dominant‐negative disease mechanism resulting from a single pathogenic sequence variant. Based on these observations, we suggest that the C19orf12 protein functions as a homo‐multimer. Further evidence in support of this hypothesis is the presence of a functional region in C19orf12 that contains a glycine‐zipper motif, which is commonly found in proteins that are known to multimerize (Gagliardi et al., 2015). Truncated C19orf12 protein variants that retain a putative interacting domain might preserve the ability to multimerize with protein from the wildtype allele yet still damage the function of the protein complex or induce degradation as a complex. In such cases, the final outcome is loss‐of‐function.

We considered haploinsufficiency as an alternate dominant disease mechanism. The location of the initiation codon methionine in several shorter transcripts (NM_001282929.1, NM_001282930.1, and NM_001282931.1) is striking, and raises the possibility of haploinsufficiency. The clustering of truncating variants, defined by the most N‐terminal of those described (p.Met76Thrfs*3 in the longer transcript NM_00103176.3 in Family 18) at the equivalent position of Met1 in the shorter transcripts, may represent the loss of protein products that perform a role in the same pathway or complexes as those encoded by the longer transcripts, but which are more sensitive to changes in protein abundance.

Five recessive families (165, 196, 248, 396, and 438, Figure 3) harbor a nonsense variant that is predicted to truncate the protein at the same codon as that occurring in dominant Family 18. All five of these families have a second pathogenic sequence variant, and none of the carrier parents shows features of MPAN. The difference between the predicted protein produced by the recessive truncation at codon 79 versus that from the dominant truncation also at codon 79 is the identity of the terminal nine amino acids. This observation suggests that the normal amino acid sequence of residues 69–76 may be necessary and sufficient for homo‐multimerization of the C19orf12 protein. Such information may inform mutation interpretation and pattern of inheritance.

### 4.6 Future directions

Functional studies are required to explore the mechanisms proposed here, including analysis of mRNA expression for NMD and the level of C19orf12 protein expression. Moreover, it will be crucial to explore whether dominant versus recessive pathogenic sequence variants differentially affect the protein‐protein interaction in order to understand the disease mechanism. Regardless, the autosomal dominant pedigrees, *de novo* cases, and clustering of similar pathogenic sequence variants in the heterozygous cohort provide sufficient evidence to support a dominant mechanism and to impact clinical care recommendations.

### 4.7 Implications for patient care

Our results directly impact clinical care of people known or suspected to have MPAN. When a single variant allele is identified in an individual with clinical features of MPAN, the clinician must consider autosomal dominant MPAN in the differential diagnosis. Our data reveal that genotype often predicts the sufficiency of a single mutation to cause disease; all dominant pathogenic sequence variants lead to a protein that is truncated after amino acid 79, with wild‐type residues 69–76 intact. As a corollary, none of the recessive sequence variants produces a protein that terminates after amino acid 79. Therefore, the precise nature and location of a sequence variant in *C19orf12* enables prediction of its sufficiency to cause disease as a heterozygous allele.

Predictive testing may be possible in individuals with a family pathogenic sequence variant but without disease signs or symptoms; however, interpretation of genetic data may be complicated by multiple factors, including incomplete penetrance and variable expressivity. Parental testing is recommended in all cases with one or two variants in order to determine phase (cis or trans) and identify whether a heterozygous *C19orf12* sequence variant in the proband is *de novo*, implicating it as likely pathogenic. Such testing will also inform recurrence risk, though gonadal mosaicism remains a possibility. Given that MPAN can present during adulthood, there may be significant reproductive implications for affected individuals with dominant disease, and their children may be at risk for MPAN. As we learn more about the function of the C19orf12 protein, new insights into disease pathogenesis will improve our care of people affected by this disorder of NBIA.

## CONFLICT OF INTEREST

Each author listed above has personally confirmed the absence of conflict of interest in relation to commercial and other relationships.
