# Supplementary material for: Autosomal dominant mitochondrial membrane protein‐associated neurodegeneration (MPAN)
Source: Mol Genet Genomic Med. 2019 May 13;7(7):e00736. doi: 10.1002/mgg3.736 (PMC6625130; doi:10.1002/mgg3.736)
Supplement: Supplementary file 2 [file MGG3-7-e00736-s002.docx]

**Supporting Information**

Video Legend

[*This patient or their legal guardians signed consent forms approved by the Baylor Institutional Board for Human Research, giving permission to be videotaped and allowing the videos to be published*.]

Video S1: Subject 18-411. The video shows the subject at age 34 years with moderate parkinsonism, manifested by mild hypomimia, bradykinesia, postural hand tremor, and decreased right arm swing.
